# Revolutionizing Healthcare: How Telemedicine Is Improving Patient Outcomes and Expanding Access to Care

**DOI:** 10.7759/cureus.63881

**Published:** 2024-07-05

**Authors:** Victor C Ezeamii, Okelue E Okobi, Hassana Wambai-Sani, Gamamedaliyanage S Perera, Shakhnoza Zaynieva, Chinwe C Okonkwo, Mohamed M Ohaiba, Pamela C William-Enemali, Okiemute R Obodo, Ngozika G Obiefuna

**Affiliations:** 1 Public Health, Jiann-Ping Hsu College of Public Health, Georgia Southern University, Statesboro, USA; 2 Family Medicine, Larkin Community Hospital Palm Springs Campus, Miami, USA; 3 Family Medicine, Medficient Health Systems, Laurel, USA; 4 Family Medicine, Lakeside Medical Center, Belle Glade, USA; 5 Family Medicine, Windsor University School of Medicine, Basseterre, KNA; 6 Family Medicine, Internal Medicine, All Saints University School of Medicine, Roseau, DMA; 7 Medicine, St. Mary Medical Center, Florida, USA; 8 Family Medicine, AdventHealth, Sebring, USA; 9 Industrial Engineering, Louisiana State University, Baton Rouge, USA; 10 Family Medicine, College of Medicine, University of Nigeria Teaching Hospital, Enugu, NGA; 11 Medicine, Windsor University School of Medicine, Cayon, KNA; 12 Pediatrics, University of Abuja Teaching Hospital, Gwagwalada, NGA

**Keywords:** telemedicine adoption, efficacy of telemedicine, application of telemedicine, access to care, telemedicine

## Abstract

Telemedicine uses digital technologies to provide healthcare services remotely, greatly improving patient access, especially during crises like the COVID-19 pandemic.

This systematic review investigates telemedicine's effects on patient outcomes, access to care, and its role in the evolving healthcare landscape. Relevant studies were identified using MeSH terms and keywords through electronic databases and manual reference list screenings. The selected empirical studies, both quantitative and qualitative, examined telemedicine across various patient populations. The review categorized findings into themes related to patient outcomes and access to care.

Telemedicine was found to be a transformative tool in chronic disease management, particularly in diabetes care. Significant improvements in patient health outcomes and cost savings were reported with telemedicine interventions. For example, telehealth platforms enhance diabetes management by increasing patient engagement and improving clinical metrics such as HbA1c levels. Additionally, telehealth services for diabetes-related foot disease (DFD) overcome geographical barriers, providing specialized care and improving patient access and satisfaction.

In conclusion, telemedicine significantly improves patient outcomes, access, and satisfaction in chronic disease management, especially diabetes care. By overcoming geographical barriers and enhancing patient engagement, telehealth platforms have the potential to transform global healthcare delivery. Implementing these insights into practice can improve the accessibility and effectiveness of diabetes care worldwide, ensuring equitable and patient-centered healthcare solutions.

## Introduction and background

The advent of telemedicine has heralded a new era in healthcare delivery, offering myriad possibilities to enhance patient outcomes and expand access to care [1]. Telemedicine, defined as using telecommunication technologies to provide and support health care at a distance, has gained substantial traction globally. Its application spans various modalities, including real-time video consultations, remote monitoring, and mobile health applications, all aimed at bridging the gap between patients and healthcare providers [2,3]. This transformation is particularly significant in the context of the COVID-19 pandemic, which acted as a catalyst for the rapid adoption and integration of telemedicine into mainstream healthcare practices [4,5].

Telemedicine’s potential to improve patient outcomes and access to care is multifaceted. It solves many long-standing challenges in healthcare, such as geographical barriers, provider shortages, and the need for timely medical intervention [6,7]. Telemedicine can eliminate the need for long-distance travel for patients in rural or underserved areas, reducing the time and costs associated with accessing healthcare. Moreover, it offers a platform for continuous monitoring and follow-up care, crucial for managing chronic diseases and improving overall health outcomes. The convenience and flexibility of telemedicine also contribute to increased patient engagement and adherence to treatment plans, as patients can schedule consultations at their convenience, potentially leading to better health management [6-10].

However, the impact of telemedicine is not uniform across all patient demographics and conditions. Factors such as digital literacy, access to technology, and socioeconomic status play a crucial role in determining the effectiveness of telemedicine services. While telemedicine can democratize access to healthcare, it also risks exacerbating existing disparities if vulnerable populations are left behind due to technological barriers. Thus, a comprehensive understanding of telemedicine's benefits and limitations is essential for policymakers, healthcare providers, and stakeholders to ensure equitable and effective implementation [11,12].

The clinical effectiveness of telemedicine in improving patient outcomes is another critical area of exploration. Studies have shown promising results in various fields, including cardiology, dermatology, mental health, and primary care [13,14]. For instance, telemedicine has been associated with improved management of chronic conditions such as diabetes and hypertension through regular remote monitoring and virtual consultations [15]. In mental health care, telepsychiatry has expanded access to mental health services, offering timely support and reducing the stigma associated with in-person visits. Moreover, integrating telemedicine into emergency care has demonstrated the potential to reduce hospital admissions and improve triage efficiency [16-18].

Despite its advantages, the implementation of telemedicine is fraught with challenges. Data security, patient privacy, and regulatory compliance are paramount concerns to ensure telemedicine services' sustainability. Additionally, the variability in telemedicine practices and standards across different regions poses a challenge for widespread adoption. There is a pressing need for standardized guidelines and best practices to optimize telemedicine delivery and ensure high-quality care [19,20].

This review aims to provide a comprehensive analysis of the impact of telemedicine on patient outcomes and access to care. By synthesizing current research and evidence, we seek to highlight the benefits and challenges associated with telemedicine, offering insights into its potential to transform healthcare delivery. Through this examination, we aim to inform future strategies and policies that can harness the power of telemedicine to enhance patient care and improve health equity. The review will also explore the implications of telemedicine for healthcare professionals (HCPs) and the necessary adaptations required to integrate telemedicine effectively into routine clinical practice.

## Review

Method

This study is a systematic review. A comprehensive literature search was carried out across multiple electronic databases to investigate how telemedicine is revolutionizing healthcare by improving patient outcomes and expanding access to care. The search strategy integrated Medical Subject Headings (MeSH) terms and keywords focusing on telemedicine, patient outcomes, and access to care. Additionally, reference lists from selected studies and pertinent review articles were manually screened to discover further eligible studies. This review included empirical studies that examined the impact of telemedicine on patient outcomes and access to care across various patient populations, using quantitative and qualitative research methods. Only peer-reviewed journal articles were included to ensure the reliability and validity of the findings.

A standardized data extraction form was utilized to gather essential information from each study, covering aspects such as study characteristics, specifics of the telemedicine intervention, measured outcomes, and key findings. Due to the diversity in study designs and outcomes, a narrative synthesis of the results was conducted. Studies were categorized based on two main themes: patient outcomes and access to care, allowing for a structured analysis of how telemedicine enhances healthcare delivery by improving patient health and broadening access to medical services.

Review of literature

The rapid expansion of telemedicine, accelerated by the SARS-CoV-2 coronavirus disease (COVID-19) pandemic, has reshaped the landscape of healthcare delivery. This review synthesizes recent findings on the impact of telemedicine on patient outcomes and access to care, drawing from a diverse array of studies conducted across various healthcare settings. Table 1 presents the findings of healthcare studies impacting telemedicine adoption and patient outcomes [21-46].

**Table 1:** Comprehensive overview of healthcare studies impacting telemedicine adoption and patient outcomes CKD, chronic kidney disease; HbA1C, blood test used in the diagnosis of type 2 diabetes; SEM, structural equation modeling; RCT, randomized controlled trial; MS, multiple sclerosis; MSFC4, multiple sclerosis functional composite; A1c, test for measuring the amount of hemoglobin with attached glucose and reflecting the average level of blood glucose in the last three months; virtualKIDS, Australia's initial pediatric-specific virtual healthcare service; ED, emergency department; UPMC, University of Pittsburgh Medical Center Health Plan; HIV, human immunodeficiency virus; PLWH, persons living with HIV; PATH, program for appropriate technology in health; COVID, coronavirus disease; SUD, substance use disorders; HCPs, healthcare professionals; DH, digital health; HNC, head and neck cancer

Sr. No.	Author name, year	Study design	Study purpose	Main finding	Conclusion
1	Belfort et al., 2024 [21]	Review of Internet technologies	Impact of Internet technologies on psychiatric practice	Internet technologies offer both beneficial and harmful outcomes, add complexities to psychiatric practice, and offer new treatment options.	Successful incorporation of digital therapeutics requires provider expertise.
2	Trần et al., 2024 [22]	Qualitative systematic review	Preferences of CKD patients on digital health interventions	Five themes: simple instruction and engaging design, individualized interventions, virtual communities of care, education and action plans, timely reminders.	Digital health interventions are important for accessing lifestyle services and should be tailored to specific needs.
3	Zakaria et al., 2024 [23]	Retrospective case-control study	Impact of remote monitoring on Type 2 diabetes	Significant HbA1c improvements (-2.19%) in case group, especially with higher baseline levels. Better outcomes with higher digital interactions.	The hybrid model of in-clinic consultations and continuous remote monitoring improves glycemic control and clinical parameters.
4	Aledia et al., 2024 [24]	Analysis of 1543 e-consults	Effectiveness of inpatient e-consult service	53.5% of requests addressed electronically, median response time of 3.7 hours for e-consults vs. 7.3 hours for in-person.	E-consults provide timely, efficient inpatient consultation services, reducing unnecessary face-to-face consultations.
5	Schmitz et al., 2024 [25]	Mixed-methods, 1200 surveys, SEM analysis	Adoption of telemedicine in Germany, Spain, and the US	Seven hypotheses (performance expectancy, hedonic motivation, habit, relative advantage, perceived security) are positive, direct, and significant.	Understanding patient perspectives can help develop strategies to promote telemedicine, improving access to care.
6	Singh et al., 2024 [26]	Retrospective data and cost analysis	Impact of IoT-driven remote monitoring on dementia patients	Reduced urgent care; significant cost savings (£201,583 annually).	Positive impact on healthcare utilization and cost avoidance.
7	Prada et al., 2024 [27]	Cross-sectional study	Economical outcomes of multidisciplinary telemedicine in Latin America	Significant travel time, distance, and cost savings.	Telemedicine significantly reduces travel costs and time.
8	Oesterle et al., 2024 [28]	Naturalistic observational study	Hybrid vs. virtual-only SUD treatment completion rates	Higher treatment completion with hybrid approach (OR: 1.88).	Hybrid model offers benefits over virtual-only SUD treatment.
9	Benda et al., 2024 [29]	Semi-structured interviews	mHealth system for Black postpartum patients	Identified relevant symptoms and design needs for mHealth.	Importance of nuanced symptom elicitation and user control.
10	McGinley et al., 2024 [30]	RCT with 120 adults, 24-month follow-up	Longitudinal impact of telemedicine on MS care	Ongoing study; primary outcome worsening in MSFC4 measures.	Study to address gaps in telemedicine for chronic conditions.
11	Li et al., 2024 [31]	Retrospective evaluation	Enhance chronic disease management via telehealth pharmacy	Increased pharmacy visits; A1c reduction observed in most patients.	Telehealth pharmacy services effective for diabetes management.
12	Toriola et al., 2024 [32]	Nursing-led telehealth service	VirtualKIDS telehealth service for pediatric non-urgent cases	44% reduction in ED visits; 69% hospitalization avoidance.	Virtual care models effective in reducing ED visits.
13	Vudathaneni et al., 2024 [33]	Prospective observational study	Telemedicine's impact on chronic disease management	Significant improvement in patient health and cost savings.	Telemedicine has transformative potential for healthcare.
14	Chen et al., 2024 [34]	Manual and framework, data from 83 patients	Digital Navigator role in mental health hybrid clinic	High satisfaction with Digital Navigator role.	Positive outcomes in digital engagement and data quality.
15	Shao et al., 2024 [35]	Retrospective cohort analysis	Impact of telemedicine on no-show visit rates	Reduced no-show rates with telemedicine, especially phone visits.	Telemedicine effectively reduces no-show visits.
16	Lynch et al, 2024 [36]	Pre-post analysis of UPMC Health Plan claims	Telemedicine bridge clinic for opioid use disorder	High buprenorphine initiation; reduced unplanned care costs.	Telemedicine bridge clinic effective for opioid use disorder.
17	Saravanakumar et al., 2024 [37]	Survey of 10 state Medicaid medical directors	Telehealth's impact on patient outcomes and access post-COVID-19	Majority support telehealth, especially video+audio. Support for remote monitoring services with guardrails.	Strong clinical endorsement for telehealth flexibility, especially video+audio, cautious about audio-only.
18	Pitpitan et al., 2024 [38]	RCT with 375 participants, follow-ups every 3 months	Efficacy of PATH intervention for HIV care among Hispanic and Black PLWH	Ongoing study, recruitment began Nov 2021, ends June 2024.	PATH intervention aims to improve HIV care engagement and viral suppression.
19	Mohanna et al., 2024 [39]	Survey of cancer center patients	Telehealth's role in mitigating COVID-19's impact on cancer care	54.1% used telehealth, 84.4% found it easy and effective.	Telehealth can be an effective tool for cancer care support during and after treatment.
20	Moulton et al., 2024 [40]	Scoping review of 100 articles	Nurse and midwife involvement in task-sharing in primary care	Nurse-led models improve health outcomes, cost-effective with training and support.	Nurse-led models are beneficial for primary care, with proper training and regulation.
21	Mondor et al., 2024 [41]	Qualitative systematic review	Virtual care's efficacy for burn patients	High patient compliance, cost-effective, improves triage.	Virtual burn care is effective for acute-phase and follow-up care.
22	Patel et al., 2024 [42]	Cohort study of 242848 Kaiser Permanente patients	Telemedicine's impact on HbA1c measurement and levels in diabetes care	Higher HbA1c testing and better HbA1c levels with early telemedicine exposure.	Telemedicine supports ongoing diabetes care and positive outcomes.
23	Queiroz et al., 2024 [43]	National cross-sectional survey	Digital health implementation among Portuguese cardiovascular HCPs	78% believe DH improves outcomes, 64% promotes health literacy, 63% reduces costs.	Positive expectations for DH in cardiovascular care, barriers include patient literacy and regulation.
24	Mali (2024) [44]	Review of digital health and telehealth interventions	Telehealth's role in HNC care	Telehealth improves physical activity, rehabilitation, and self-management.	Telehealth increases access to care and continuity of services for cancer patients.
25	Garg et al., 2024 [45]	Cross-sectional study in a tertiary care setup	Telemedicine's role in patient satisfaction and engagement	Improved satisfaction scores, better medication adherence, reduced ED times.	Telemedicine is essential for healthcare delivery post-COVID-19.
26	Graham, 2024 [46]	Qualitative study with 11 Aboriginal participants	Telehealth service for diabetes-related foot disease management	Reduced travel burden, improved access to specialists, enhanced communication.	Telehealth facilitates trust, safety, and better access to specialist care.

Enhanced chronic disease management

The efficacy of telemedicine in managing chronic diseases is well-supported by numerous studies [21-23,30-33,39,40,46]. For example, a retrospective evaluation of telehealth pharmacy services revealed increased pharmacy visits and A1c (blood test for diagnosis of type 1 and type 2 diabetes) reductions among most patients, indicating effective diabetes management through telehealth platforms [31]. Furthermore, a prospective observational study involving 186 participants demonstrated significant improvements in patient health and cost savings due to telemedicine interventions, with notable increases in patient satisfaction and accessibility [33].

Mental health hybrid clinics

Belfort et al. have explored how Internet technologies impact children, adolescents, and psychiatric practice, revealing a nuanced landscape of benefits and challenges. While these technologies enhance patient accessibility, they also introduce complexities into psychiatric care, requiring specialized knowledge for the effective integration of digital therapeutics. The study highlights issues such as navigating electronic health records (EHRs), managing patient portals, and maintaining meaningful virtual patient interactions [21]. In a study focused on short-term cognitive-behavioral therapy, researchers introduced the Digital Navigator role within a hybrid clinic to enhance patient engagement and satisfaction, as well as digital phenotyping data quality. Results from 83 patients indicated high satisfaction with Digital Navigators, unaffected by demographic factors. Analysis of application engagement and passive data quality among 33 patients further supported the role's effectiveness, showing positive correlations with patient satisfaction [34].

These findings highlight the potential of Digital Navigators to enhance care access, improve patient adherence, and alleviate clinician workflow burdens. Standardizing this role could facilitate broader adoption in mental health care settings, promoting scalable and effective use of digital technologies to support patient mental health outcomes.

Preferences of CKD patients regarding digital health interventions

This review underscores the significance of digital health interventions in facilitating access to lifestyle services for individuals with chronic kidney disease (CKD). By synthesizing consumers' preferences, including the need for simple, engaging designs, personalized approaches, virtual support communities, educational resources, and automated monitoring, it highlights key considerations for developing effective interventions. Tailoring digital tools to these preferences is essential for enhancing engagement and adherence among CKD patients, potentially improving health outcomes. Future research should focus on implementing these insights to design and assess the impact of targeted digital interventions on health behaviors and overall CKD management [22].

Advancements in telemedicine for diabetes management

Telemedicine has emerged as a pivotal tool in managing diabetes, as evidenced by several recent studies. Digital interactions were positively correlated with reductions in both HbA1c (blood test for diagnosis of type 2 diabetes) and weight, suggesting that the hybrid approach may offer superior outcomes compared to digital-only interventions [23]. A cohort study involving 242848 Kaiser Permanente patients demonstrated that early exposure to primary care telemedicine (telephone or video) was associated with significantly higher rates of HbA1c measurement (91.0% for video, 90.5% for telephone vs. 86.7% without visits) and lower HbA1c levels (<8%: 68.5% video, 67.3% telephone vs. 66.6% without visits). This study underscored telemedicine's role in enhancing patient engagement and improving clinical outcomes during the SARS-CoV-2 coronavirus disease (COVID-19) pandemic [44]. Another retrospective case-control study focused on type 2 diabetes patients emphasized the efficacy of a hybrid model combining in-clinic consultations with continuous remote monitoring. This approach led to significant improvements in HbA1c levels, particularly among patients with higher baseline levels. Furthermore, telehealth has proven beneficial in managing diabetes-related foot disease (DFD), which accounts for a substantial portion of global lower-extremity amputations with high post-amputation mortality rates. Rural and Indigenous populations face disproportionate challenges due to limited access to specialized care. At the Royal Adelaide Hospital, a telehealth service utilized real-time video consultations to overcome geographical barriers for Aboriginal and Torres Strait Islander communities. Qualitative findings highlighted reduced travel burdens, improved access to specialists, and enhanced patient reassurance and communication through virtual specialist consultations. This approach not only supports effective clinical management but also fosters trust and patient well-being by providing personalized care remotely [46].

Multiple sclerosis

McGinley et al. explored the long-term effects of telemedicine on multiple sclerosis (MS) management [30]. Their study compares in-clinic and telemedicine approaches over a 24-month period, evaluating clinical outcomes, economic implications, patient satisfaction, and treatment adherence. The findings highlight telemedicine's potential to improve access and patient experiences while maintaining effective clinical outcomes. These insights can guide future strategies in MS care and clinical trial designs, emphasizing telemedicine's role in reducing healthcare delivery barriers and enhancing patient outcomes [30].

Transforming cancer care: digital health innovations

Digital healthcare, encompassing telemedicine and telemonitoring, shows promise in addressing challenges posed by increasing head and neck cancer (HNC) cases and healthcare shortages. Multidisciplinary tele-practice models, including speech-language pathology, enhance HNC management. Mobile health interventions aid in physical activity and rehabilitation for cancer survivors. Telecommunication advancements support remote interventions like swallowing exercises, education, and symptom monitoring. Amid the COVID-19 pandemic, home-based remote rehabilitation gains urgency, though optimal strategies remain evolving. Telehealth offers a novel approach across the cancer continuum, bolstering patient self-management, knowledge, and access to care, despite disparities skewed toward younger, English-speaking, and female patients, exacerbated by pandemic impacts [44]. Similarly, during the COVID-19 pandemic, telehealth effectively mitigated its impact on cancer care, with 54.1% of patients at an academic cancer center using telehealth and 84.4% finding it both easy and effective [39].

Digital health in cardiovascular medicine

A national survey among Portuguese cardiovascular HCPs reveals widespread use and high expectations for digital health tools. Most participants (78%) anticipate digital health to enhance health outcomes, while barriers like patient technology literacy and regulatory gaps hinder broader adoption. Smartphone, laptop, and tablet ubiquity among respondents underscores DH's potential to transform cardiovascular care, emphasizing the need for enhanced patient education and regulatory frameworks to optimize digital health integration [43].

Impact on specific populations

Dementia and Substance Use Disorders

Telemedicine has demonstrated significant benefits for patients with dementia and substance use disorders. For instance, an analysis of IoT-driven remote monitoring for community dementia patients showed a reduction in urgent care visits and considerable cost savings, highlighting positive impacts on healthcare utilization and cost avoidance [26]. Additionally, for substance use disorders, a naturalistic observational study found higher treatment completion rates with a hybrid approach compared to virtual-only treatment, suggesting enhanced effectiveness through a combination of in-person and digital interventions [28].

We found that the telemedicine bridge clinic effectively initiated buprenorphine treatment and facilitated ongoing care for patients with opioid use disorder. Results showed high rates of buprenorphine prescription fills and significant reductions in unplanned care costs, medical costs, and overall healthcare costs. This approach led to increased utilization of primary care and outpatient behavioral health services, while emergency department (ED), urgent care, and inpatient costs decreased. The findings support the telemedicine bridge clinic model as beneficial for improving treatment outcomes, reducing healthcare expenditures, and enhancing patient care continuity [36].

Black Postpartum Patients

Benda et al. (2024) address the critical need for patient-centered mobile healthcare (mHealth) solutions to monitor postpartum symptoms among Black patients, aiming to mitigate disparities in healthcare outcomes. Their proposed mHealth system incorporates somatic and psychological symptom inputs while considering various sociocultural and systemic factors. This approach empowers patients to make informed decisions about seeking medical care. Key recommendations for system design emphasize user control, clear and supportive messaging, and integration with broader supportive resources. Future efforts should focus on creating practical, accessible mHealth tools that cater to diverse patient needs in perinatal and other clinical settings [29].

Pediatric Non-urgent Cases

During the COVID-19 pandemic, virtualKIDS (pediatric-specific virtual healthcare service), a nursing-led telehealth service in New South Wales, Australia, effectively managed pediatric non-urgent cases, reducing the strain on EDs. Operating 24/7 with features like virtualKIDS Acute Response (vKAR) and Virtual Urgent Care (VUC), the service saw a notable decrease in ED visits by 44% within 48 hours and a 69% decrease in hospital admissions. Through nursing-led triage and audiovisual consultations, virtualKIDS demonstrated its capacity to enhance accessibility and outcomes in pediatric care. Innovative approaches such as home sleep studies and day-only surgeries further underscore its potential as a valuable alternative to traditional acute-care settings [32].

Efficacy of PATH intervention for HIV care

The PATH trial integrates peer navigation with a mHealth app to enhance human immunodeficiency virus (HIV) care engagement and viral suppression among Hispanic and Black persons living with HIV (PLWH). This approach aims to address disparities in HIV outcomes by leveraging scalable technology and peer support. By evaluating sustained viral suppression and secondary outcomes like retention in care and ART adherence, the trial seeks to elucidate the intervention's impact and implementation feasibility within Ryan White Program settings. Results will contribute valuable insights into optimizing HIV care delivery for underserved populations, potentially enhancing long-term health outcomes in these communities [38].

Impact and integration of virtual care for burn injuries

Virtual care for burn injuries varies in impact. This review assesses its efficacy, costs, and patient outcomes via PRISMA-compliant qualitative systematic review methods. Analyzing 481 studies, 37 were included, mainly observational. Virtual care benefits acute and outpatient phases, enhancing specialist access and patient compliance. Challenges include information technology (IT) issues and privacy concerns. Evidence suggests moderate effectiveness, cost-efficiency, and improved triage, supporting virtual burn care's integration into routine and acute care settings [41].

Efficiency in inpatient consultation services and no-show rates reduction

The efficiency of telemedicine in providing inpatient consultation services has been validated through various analyses. One study analyzing 1543 inpatient e-consults across 11 specialties found that 53.5% of requests were addressed electronically, with a median response time of 3.7 hours for e-consults compared to 7.3 hours for in-person consultations. This efficiency in providing timely and effective consultation services reduces unnecessary face-to-face consultations and improves inpatient care coordination, particularly during COVID-19 surges [24]. Economic outcomes of multidisciplinary telemedicine service in Latin America: Prada et al. (2024) highlight significant economic benefits of telemedicine implementation in a high-complexity hospital in Latin America, particularly through substantial savings in travel costs and time for patients across different geographical regions. These findings underscore telemedicine's potential to mitigate barriers to healthcare access, particularly in regions facing geographic and economic challenges. The results advocate for continued investment and expansion of telemedicine services in similar settings to enhance healthcare delivery efficiency and equity, ultimately improving patient outcomes and reducing healthcare disparities [27].

Telemedicine has proven effective in reducing no-show rates for medical appointments. A retrospective cohort analysis of clinic visits from 2020 to 2023 revealed that telemedicine, particularly phone visits, significantly reduced no-show rates. This underscores the importance of including phone visits in the definition of telemedicine to maximize its benefits in patient engagement and care continuity [35].

Telemedicine adoption and patient perspectives

Understanding patient perspectives on telemedicine is crucial for its adoption and efficacy. A mixed-methods approach involving 1200 surveys across Germany, Spain, and the US identified seven positive, direct, and significant factors, including performance expectancy, hedonic motivation, habit, relative advantage, and perceived security. The study concluded that comprehending patient perspectives can help develop strategies to promote telemedicine, thereby improving access to care [25]. Additionally, a survey of 10 state Medicaid medical directors indicated strong clinical endorsement for telehealth flexibility, particularly for video/audio services, while emphasizing the need for guardrails and careful consideration of fiscal impacts and technical implementations [37]. Nurse-led task-sharing models offer a promising approach to improving health equity and accessibility in primary care. Despite observed positive outcomes, the literature identifies gaps in detailing the implementation and operational mechanisms of these models within primary care settings. Recommendations include scaling up nurse-led initiatives with improved training, financial support, and regulatory alignment to maximize their feasibility and effectiveness on a global scale [40]. During the COVID-19 pandemic, telemedicine emerged as a crucial tool, utilizing digital technologies to bridge distances and deliver healthcare remotely. A cross-sectional study demonstrated significant improvements in patient satisfaction with telemedicine. Enhanced medication adherence, reduced ED visits, and strong correlations between readmission rates and medication compliance were observed, underscoring telemedicine's pivotal role in modern healthcare delivery. These findings highlight its potential to persist post-pandemic and integrate effectively into existing healthcare systems to enhance patient outcomes and operational efficiencies [45].

Key recommendations for telemedicine optimization and equity

Telemedicine models should include both video and phone visits to fully realize their benefits. Hybrid models that combine in-person and telehealth services often yield better outcomes than digital-only approaches. Continuous evaluation and adaptation are necessary to address disparities in telehealth usage across different age groups, languages, and genders. The role of digital navigators can enhance patient engagement and data quality, and standardizing their role could benefit broader clinical use. Further research is needed to explore the dynamics of in-person components in hybrid treatment models and validate findings in larger cohorts. Telemedicine models should prioritize inclusivity and flexibility, incorporating both video and phone visits to accommodate diverse patient needs. Continuous evaluation and adaptation are essential to address technological challenges and disparities in telehealth usage, ensuring equitable access to care for all patient populations.

Strengths and limitations

The study's strength lies in its comprehensive synthesis of recent findings, demonstrating telemedicine's transformative impact on healthcare delivery. Across diverse healthcare settings, it effectively highlights telemedicine's efficacy in managing chronic diseases like diabetes, significantly improving patient outcomes and satisfaction. The review includes studies showcasing hybrid models, combining in-clinic consultations with remote monitoring, which underscore telemedicine's versatility and effectiveness. Furthermore, its exploration of telemedicine's impact on specific populations, such as those with dementia, cancer, and substance use disorders, underscores its broad applicability in addressing diverse healthcare needs. However, limitations exist within the study. Potential biases inherent in the included research, such as selection bias or publication bias, may limit the generalizability of findings. Some studies relying on retrospective data analysis or self-reported measures could introduce inaccuracies or recall biases, affecting conclusion validity. Methodological heterogeneity and diverse study populations pose challenges in directly comparing findings or extrapolating overarching trends. Moreover, the rapid evolution of telemedicine practices, particularly post-COVID-19, may render some included research outdated or less relevant. Finally, while the review emphasizes telemedicine's benefits, it may not fully capture potential negative consequences, such as disparities in access or concerns regarding data privacy and security associated with its implementation.

## Conclusions

The findings in this review summarise several key things. First, the rapid expansion of telemedicine, catalyzed by the COVID-19 pandemic, has profoundly reshaped healthcare delivery, notably in chronic disease management and patient access. Second, this study demonstrates that telemedicine alone, and also in addition to hybrid model-integration in-clinic consultations with remote monitoring, has proven highly efficient in enhancing glycemic control and reducing healthcare costs. Furthermore, addressing no-show rates through telemedicine, particularly via phone visits, enhances patient engagement and continuity of care. In addition, telemedicine's efficiency in inpatient consultations has streamlined care delivery while minimizing face-to-face interactions during peak pandemic periods. Also, patient perspectives, including performance expectancy and security concerns, may influence telemedicine adoption and satisfaction. Finally, telemedicine's impact spans diverse populations, from dementia patients to cancer care and substance use disorders, yielding reduced urgent care visits and improved treatment adherence. These findings support telemedicine's pivotal role in transforming healthcare delivery, improving outcomes, and expanding access to care across varied settings and populations, ensuring sustained benefits post-pandemic.
